# Integrated traditional Chinese medicine improves acute pancreatitis via the downregulation of PRSS1 and SPINK1

**DOI:** 10.3892/etm.2015.2191

**Published:** 2015-01-20

**Authors:** QIANG GAO, NUSHENG LIANG

**Affiliations:** 1 Physician Department, Health Service Center, Nyalam, Tibet 858300, P.R. China; 2 Gastroenterology Department, Yantai Hospital of Traditional Chinese Medicine, Yantai, Shandong 264002, P.R. China; 3 Department of Gastroenterology, First Hospital of Tianjin, Tianjin 300193, P.R. China

**Keywords:** integrated traditional Chinese medicine, acute pancreatitis, protein, serine 1, serine peptidase inhibitor, Kazal type 1

## Abstract

Integrated traditional Chinese medicine (ITCM) is known to improve health in patients with acute pancreatitis (AP); however, the molecular mechanisms underlying this effect are unknown. AP is associated with the expression of PRSS1 and SPINK1. Thus, the present study aimed to investigate whether ITCM was able to ameliorate AP by regulating the expression levels of protein, serine 1 (PRSS1) and serine peptidase inhibitor, Kazal type 1 (SPINK1). A total of 100 AP patients were divided at random into two groups. The treatment group were treated externally with a herbal ITCM preparation, while the control group received a routine placebo treatment. The mRNA and protein expression levels of PRSS1 and SPINK1 were subsequently compared between the two groups. The results revealed that the health of the patients who had received ITCM improved significantly when compared with the control group patients (P<0.05). In addition, the expression levels of PRSS1 and SPINK1 were found to be lower in the treatment group when compared with the control group (P<0.05). Therefore, ITCM exhibited a significant therapeutic effect on AP and produced no side effects since the treatment was applied externally. ITCM may ameliorate AP by downregulating the expression of PRSS1 and SPINK1; thus, should be considered as a potential therapy for the development of drugs against AP.

## Introduction

Acute pancreatitis (AP) is a common disease of the digestive system, characterized by acute, severe symptoms, a variety of complications and a high rate of mortality (1,2). Paralytic intestinal obstruction is an early clinical symptom of AP, occurring in >80% of cases (3). The pathogenesis of pancreatitis is considered to be associated with the inflammatory response and an abnormal immune reaction (4,5), which are caused by the activation of pancreatic enzymes, fermentemia and numerous cytokines and inflammatory mediators (6). The intestinal hypomotility caused by the pathogenesis of pancreatitis may therefore lead to AP (7). In the course of AP, the intestinal mucosa barrier is damaged by the translocation of intestinal bacteria and endotoxins (8–10). AP aggravates the inflammatory reaction, interferes with the nerve function of the gastrointestinal tract, compromises blood circulation in the intestines and damages the intestinal mucosa. This damage may result in an intestinal motility disorder, flatulence, bowel dilatation and abdominal pressure, which may in turn affect the gastrointestinal blood supply and increase microcirculation (11,12). In addition, the large volume of liquid exudation, damage to the intestinal mucosal barrier, bacterial translocation and the absorption of toxins may aggravate shock and systemic infection, resulting in mortality (13,14). There is a close association between AP and infective pancreatic necrosis (15,16).

However, the etiology of AP is complicated and the appropriate therapy for the disease remains contested (2,17). The application of acupuncture, when combined with Western medicine, has been considered an effective therapy for AP (18). The majority of treatments for AP have evident side effects (19), which limit their use in the treatment of AP. Integrated traditional Chinese medicine (ITCM) has been practiced for over 1,000 years and is widely used as an external treatment for AP, with few side effects (20–22). The present study reports a novel ITCM treatment that is an effective therapy for AP. However, the mechanism underlying the effect of ITCM in the treatment of AP remains unknown. The protein, serine 1 (PRSS1) gene, which expresses cationic trypsinogen, is associated with hereditary pancreatitis and characterized by acute inflammation of the pancreas (23). SPINK1, which codes for serine protease inhibitor Kazal-type 1, is an additional gene regarded as a disease modifier of AP (24,25). Thus, AP has been shown to be associated with the expression of PRSS1 and serine peptidase inhibitor, Kazal type 1 (SPINK1) (24,26,27). The aim of the present study was to investigate whether ITCM may ameliorate AP by regulating the expression of PRSS1 and SPINK1.

## Materials and methods

### Participants

The study protocols on human subjects and consent documents were approved by the Human Research Ethics Committee of Yantai Hospital of Traditional Chinese Medicine (Yantai, China). In addition, written consent documents were obtained and the data were collected with a Certificate of Confidentiality protecting patient privacy. A total of 100 AP patients were recruited from the Yantai Hospital of Traditional Chinese Medicine between March 2009 and March 2014 in order to rule our or prevent any genetic differences. All patients were unrelated, ethnic Han Chinese with similar cultural and economic backgrounds.

AP patients were divided at random into two groups (Table I). In total, 100 patients (age range, 40–55 years) were examined who were reported to be suffering with AP, but had no symptoms of other intestinal or inflammatory diseases. All eligible cases had their diagnoses confirmed by histological examination. The course of the disease ranged between 3 h and three days. Of the 100 cases, 48 were biliary in origin, 40 cases were due to overeating and overdrinking and 12 cases were the result of unidentified causes. In 94 cases, the patient was suffering from AP for the first time, while the remaining six cases were recurrent.

### Diagnostic criteria and disease classification

The diagnostic criteria for AP patients were in accordance with a previous study (28), and were based upon the elevated levels of pancreatic enzymes in the blood and urine. According to the diagnostic criteria, patients presenting with the following symptoms were considered to suffer from AP: Upper abdominal pain; enhanced levels of lipase, examined using a Human Pancreatic Lipase ELISA kit (Wuhan Jiacheng Biotechnology Co., Ltd., Wuhan, China); ultrasonography (US) scans (GE LOCIQ 400; GE Healthcare, Milwaukee, WI, USA) demonstrating dilation of the common bile duct or swelling of the pancreas. Clinical manifestations of AP included sudden and sharp cutting pains in upper abdomen, nausea and vomiting, fever, several days without defecation, abdominal distension, dry and bitter taste in the mouth, coated and dry or burned black tongue and forceful sphygmus. All other abdominal diseases associated with pancreatitis were ruled out. In addition, peripheral blood tests revealed increases in the number of peripheral white blood cells, neutrophils and serum amylase, while an increase in the level of urine amylase was also observed. Diffuse enlargement of the pancreas was observed in B-mode US and computed tomography (CT) examinations. The bioactivities of serum and urine amylase were analyzed in accordance with procedures described in previous studies (29,30). Blood lipase levels were also measured, since lipase is a more sensitive and specific indicator of AP compared with other pancreatic enzymes. CT scanning is the preferred method for confirming the image findings of AP.

AP can be classified as mild, moderate or severe, according to the disease severity (31). Mild AP patients exhibit no organ failure, nor any local or systemic complications. Moderate AP is characterized by the presence of transient organ failure, while severe AP patients exhibit persistent organ failure for >48 h.

### Therapeutic methods

Experimental group patients were externally administered with an ITCM preparation. The ITCM preparation was composed and prepared in accordance with previous studies (32,33) as follows: 30 g Chinese rhubarb, 15 g *Citrus aurantium*, 15 g magnolia bark, 10 g mirabilite, 10 g *Pinellia ternata*, 10 g *Coptis chinensis*, 15 g *Scutellaria baicalensis*, 10 g *Gardenia*, 15 g Radix Paeoniae Alba, 15 g tree peony bark, 15 g Radix Paeoniae Rubra, 15 g Rhizoma Corydalis, 10 g *Bupleurum* and 10 g licorice. The materials were ground to a powder using a high-pressure homogenizer (Jiangsu Makwell Machinery Co., Ltd., Huai’an, China). These powders were dissolved in 500 ml water with 200 g starch, and the mixture was concentrated and dried to form pastes of ~5×5×1 cm. The pastes were applied to the acupuncture points (18,34), Yishu, Zhongwan, Tianshu, Zusanli, Guan Yuan and Pishu, twice daily. The dose of rhubarb and mirabilite was adjusted according to the severity of the patients’ abdominal distention and pain. In addition, the composition of the powder was modified according to patient symptoms, tongue coating and pulse. The control group patients received acupuncture point application with a placebo treatment, containing a mixture of cornmeal, starch and flour.

### Curative standard

A curative standard was defined in order to categorize the efficacy of the ITCM treatment. The standard was defined as the regression of symptoms, including abdominal pain and distension, fever, vomiting and passing of feces or wind. Furthermore, improvements to the patient abdominal muscle tension, abdominal tenderness, rebound tenderness and bowel sound were considered to indicate successful therapy. In addition, blood and urine amylase levels, and routine blood and liver function tests, were returned to normal, and the extent of pancreatic edema had improved when compared with the previous abdominal B-mode US and CT examinations. The following categories were used for defining the therapeutic results: Excellent, reaching the aforementioned standard in four days; effective, reaching the aforementioned standard in seven days; inefficient, failing to reach the standard after eight days.

### Sample isolation

Sinker-assisted endoscopic submucosal dissection (ESD) is an effective technique for the removal of minus superficial pancreatic tissues (35); thus, this technique is the preferred approach for obtaining surgical specimens from AP patients. Ethical issues were considered to be very important. It was a priority to ensure that patient participation was entirely voluntary and that patient privacy was maintained. Experts conducting the study were not to attempt to persuade participants to donate any tissues if the participant was at all in doubt regarding the safety or ethics of the study.

### Potential mediating factors

A number of confounding factors have been reported to be associated with an increased risk of AP (36–40). A previous meta-analysis indicated that obesity was associated with a higher risk of AP (41); therefore, the body mass index (BMI) of the patients was measured in the present study. Gender has also been regarded as an important determinant of outcome in AP patients (42); thus, the data for the relative female and male reactions to AP treatment were also surveyed. Alcohol consumption can increase the risk of AP (43,44); therefore, all the subjects were non-drinkers, having never consumed alcohol, in order to avoid interference. To avoid age-related issues (45,46), all individuals were aged between 40 and 55 years-old. All volunteers reported a food consumption frequency of three meals per day, and the food consumption was consistent to a healthy Nordic food index (47,48). Calorie intake was calculated according to self-reported assessment of daily calorie intake (49).

### ELISA

The concentration of lipase was examined using an ELISA, with lipase antibodies purchased from Wuhan Jiacheng Biotechnology Co., Ltd. (Wuhan, China). Biopsy specimens were ground using liquid nitrogen. The grounded specimens were diluted by 1/200 in phosphate-buffered saline (PBS) and transferred to antibody-coated wells. Following the manufacturer’s instructions, all wells were washed three times with PBS, and IgG (Immuno Pure^®^ peroxidase-conjugated goat anti-human IgG; 21348; Beijing Jiamay Biotechnology Ltd., Beijing, China) was added and incubated for 30 min. After washing, 3,3′,5,5′-tetramethylbenzidine (54827-17-7; Robiot Co., Ltd., Nanjing, China) was added and cultured for 10 min for visualization. Finally, the absorbance of the mixture was examined at 450 nm using a SM600 ELISA plate reader (Shanghai Utrao Medical Instrument Co., Ltd., Shanghai, China). The concentration of lipase was calculated according to a standard calibrator curve.

### Quantitative reverse transcription polymerase chain reaction (qRT-PCR)

The mRNA expression levels of PRSS1 and SPINK1 were assessed using qRT-PCR. RNA was extracted from the AP tissues using a GenElute™ Mammalian Total RNA Miniprep kit (RTN10; Sigma-Aldrich Trading Co., Ltd., Shanghai, China). Initial cDNA was amplified from RNA using a random primer. The cDNA molecules were used as a template for qRT-PCR of the PRSS1 and SPINK1 transcripts on a rapid qTOWER 2.0 (Analytik Jena AG, Thuringia, Germany). The primers for qRT-PCR were as follows: PRSS1 (GenBank no. BC103998.2) sense, 5′-AGGGGAATGAGCAGTTCATC-3′ and antisense, 5′-CACCAGAACTCAGAGTGTTG-3′; SPINK1 (GenBank no. NM003122.3), sense, 5′-GAAGAGACGTGG TAAGTGCG-3′ and antisense, 5′-CCATCAGTCCCACAG ACAGGG-3′; GAPDH sense, 5′-CCCTTCATTGACCTCAAC TAC-3′ and antisense, 5′-CCACCTTCTTGATGTCATCAT-3′. GAPDH was used as an internal control. All genes were amplified by 30 cycles of heating for 30 sec at 94°C, followed by 1 min at 60°C. The quality of the synthesized cDNA was determined using GAPDH as the reference gene.

### Protein expression levels of PRSS1 and SPINK1

Protein expression levels of PRSS1 and SPINK1 were determined by western blot analysis. The protein was isolated from selected AP tissues and separated using 12% SDS-PAGE. The separated protein was electrotransferred onto polyvinylidene fluoride membranes (Bio-Rad Laboratories, Inc., Hercules, CA, USA), which were blocked with 5% non-fat dry milk for 1 h, and then incubated with a primary antibody: Mouse anti-human PRSS1 monoclonal antibody (MAB3848; 1:5,000; R&D Systems China, Shanghai, China) and mouse anti-human SPINK1 monoclonal antibody (70R-5308; 1:2,000; Beijing Dakewei Bio-Technology Co, Ltd., Beijing, China). A secondary antibody (anti-mouse horseradish peroxidase-conjugated; cat. no. 201201; 1:10,000; Shanghai Guoyuan Biotechnology Co., Ltd., Shanghai, China) was added and chemiluminescence detection performed using an kit from GE Healthcare (Piscataway, NJ, USA).

### Statistical analysis

The t-test and the χ^2^ test were used to detect the statistical significance for the variables between the experimental and control groups. Data were analyzed using SPSS 13.0 software (SPSS, Inc., Chicago, IL, USA), and P<0.05 was considered to indicate a statistically significant difference.

## Results

### Characteristics of AP patients

A total of 100 AP patients (64 male, 50.8±4.2 years; 36 female, 47.5±7.5 years) were recruited. Considering the complicated causative factors for increasing the risk of AP, the gender, age, BMI and daily calorie intake of the patients were not contributing factors (Table I; P>0.05). All these factors were excluded between the experimental and control groups during the data collection. The number of cases of each stage of AP were similar between the experimental and control groups (Table I).

### Effects of ITCM on AP patients

Effects of ITCM on the health outcomes of AP patients were investigated, as shown in Table I. All AP patients were diagnosed by gastroenterologists. Following eight days of treatment with ITCM, 29 cases (26 mild, two modest and one severe case) exhibited excellent results, 17 cases (11 modest and six severe) showed an effective response and four cases (severe) exhibited inefficient results. In the control group, one patient exhibited excellent results, two cases showed an effective outcome, while all the other cases resulted in an inefficient outcome. The excellent and effective outcomes in the control group were hypothesized to be the result of natural recovery since the onset of AP. The results demonstrated that ITCM showed strong therapeutic results for AP. In addition, all the symptoms of AP in the experimental group patients were improved significantly when compared with those in the control group (P<0.05; Table II). The results suggested that ITCM was able to ameliorate AP; thus, ITCM should be considered as a potential drug candidate for the treatment of AP.

### mRNA expression levels of PRSS1 and SPINK1 in AP patients

**Figure 1:**
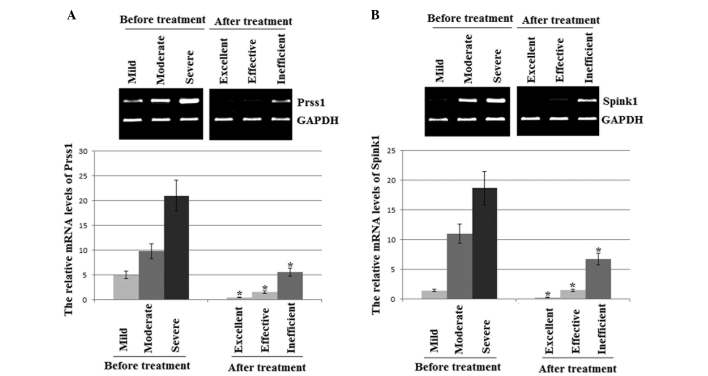
mRNA expression levels of (A) PRSS1 and (B) SPINK1 in AP patients before and after treatment. Patients were divided into three groups based on the severity of their AP; mild, modest and severe. Out of a total of 50 AP patients that underwent ITCM therapy, 26 exhibited mild AP (light grey), 13 exhibited modest AP (medium grey) and 11 patients had severe AP (dark grey). Following ITCM treatment, 29 patients exhibited excellent therapeutic results (26 mild, two modest and one severe), 17 patients exhibited effective results (11 modest and six severe) and four severe patients showed an ineffective response. Data are represented as the mean ± standard deviation of three independent experiments. ^*^P<0.01, vs. value before treatment. AP, acute pancreatitis; ITCM, integrated traditional Chinese medicine.

The qRT-PCR results revealed that the mRNA expression levels of PRSS1 and SPINK1 were lower in the AP patients treated with ITCM than those prior to treatment. The levels of PRSS1 and SPINK1 were lowest in the excellent outcome experimental group patients, moderate in the effective result patients and highest in the control and inefficient result patients (Fig. 1).

### Protein expression levels of PRSS1 and SPINK1 in AP patients

**Figure 2:**
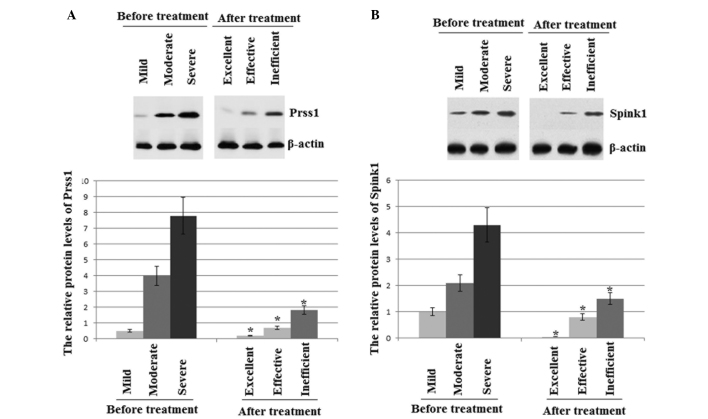
Protein expression levels of (A) PRSS1 and (B) SPINK1 in AP patients before and after treatment. Patients were divided into three groups based on the severity of their AP; mild, modest and severe. Out of a total of 50 AP patients that underwent ITCM therapy, 26 exhibited mild AP (light grey), 13 exhibited modest AP (medium grey) and 11 patients were diagnosed with severe AP (dark grey). Following ITCM treatment, 29 patients exhibited excellent therapeutic results (26 mild, two modest and one severe), 17 patients exhibited an effective outcome (11 modest and six severe) and four severe patients showed an ineffective response. Data are represented as the mean ± standard deviation of three independent experiments. ^*^P<0.01, vs. value before treatment. AP, acute pancreatitis; ITCM, integrated traditional Chinese medicine.

Levels of PRSS1 and SPINK1 proteins are known to be closely associated with the development of AP. The protein expression levels of PRSS1 and SPINK1 were at their highest prior to treatment with ITCM (Fig. 2). Following treatment with ITCM, the protein expression levels of PRSS1 and SPINK1 were lowest in the excellent outcome patients, moderate in the effective outcome patients and highest in the control and inefficient result patients (Fig. 2). Thus, the results were consistent with a previous study (50), which reported that PRSS1 and SPINK1 were potential negative biomarkers for the diagnosis and prognosis of AP.

## Discussion

AP is the most common intestinal disease worldwide, with a mortality rate as high as 36–50% for severe AP (20). Severe AP can cause serious inflammation in other systems and patients may succumb suddenly to the disease (14,51). Significant progress has been reported in the treatment of AP by ITCM (21,52,53); however, there remain a number of difficulties that hinder the improvement of therapeutic efficacy. TCM methods are commonly used in an integrative manner; thus, the present study aimed to assess the efficacy of an ITCM approach in the therapy of AP.

However, the molecular mechanisms underlying the pathogenesis of AP are not yet known, and a suitable biomarker for AP must be identified in order to further investigate these mechanisms. In certain cases, the levels of a number of important proteins are undetectable in the serum, which may be affected by the instability of human clinical and metabolic conditions. Thus, only newly obtained AP specimens were considered for examination in the present study.

Mutations in the human PRSS1 gene are associated with pancreatitis and have provided insight into the pathogenesis of the disease (54). PRSS1 is widely reported to be associated with AP (27,55,56). In addition, SPINK1 was originally identified as a trypsin inhibitor and is strongly elevated in patients with pancreatitis, where the level of elevation correlates with the disease severity (57). This association between SPINK1 and AP has also been widely reported (24,26,58–63). Thus, the present study aimed to investigate whether the expression levels of PRSS1 and SPINK1 were closely associated with the development of AP.

The sinker-assisted ESD approach to sampling AP patient tissues facilitated the detection of pancreatitis by examination of mRNA levels. The mRNA expression levels were examined initially, following which the protein levels were determined. The mRNA and protein expression levels of PRSS1 and SPINK1 demonstrated the same changed trend with the development of AP (Fig. 1). Therefore, PRSS1 and SPINK1 are potential combined adjuvant biomarkers for investigating the mechanisms underlying the increased risk of AP, and PRSS1 and SPINK1 should be considered as targets for drug therapy. Furthermore, the protein expression levels of PRSS1 and SPINK1 were lower on average in the experimental group when compared with the control group (Fig. 2). Therefore, the development of AP can be characterized by the levels of predominant PRSS1 and SPINK1. Since the expression levels of PRSS1 and SPINK1 were higher in the AP patients of the control group, the changing levels of these biomarkers may be the result of inflammatory processes. The levels of PRSS1 and SPINK1 in the AP patients treated with placebos were comparable to the levels prior to treatment (data not shown).

The application of Chinese herbs to acupuncture points is a type of external TCM. The treatment involves processing the herbs into paste, powder or ointment and subsequently applying them to corresponding acupuncture points (18,34). The interactions among the drugs, main and collateral channels and acupuncture points are involved in the treatment of AP. Medicine is absorbed through the skin, but not the digestive system; thus, the procedure is simple and safe to conduct, with no risk of an adverse reaction. ITCM has been clinically observed to ameliorate a variety of symptoms and conditions, including pain, abdominal distension, constipation, inflammation, endotoxin absorption, enterogenic infection and intestine failure. In addition, ITCM has been shown to improve the levels of amylase in the blood and urine, improve pancreatic blood circulation, promote the absorption of necrotic tissue and gastrointestinal peristalsis and decrease a variety of complications, improving the overall prognosis (64).

A number of important questions should be considered in future studies investigating the effects of ITCM in the treatment of AP. Firstly, the expression levels of PRSS1 and SPINK1 should be assessed in healthy control subjects to better understand the mechanism underlying the therapeutic effects of ITCM. However, it is difficult to recruit appropriate volunteers as the majority of healthy subjects are wary of the potential side effects of surgery based on sinker-assisted ESD techniques. Secondly, the classification system for the various stages of AP requires improvement via the design of a more precise scale.

In conclusion, the present study examined 100 AP patients in order to investigate the effects of ITCM therapy on AP. The results revealed that the development of AP was positively associated with the expression levels of the biomarkers, PRSS1 and SPINK1. Thus, PRSS1 and SPINK1 may be useful combined targets for the treatment of AP. ITCM produced significant therapeutic results for AP when compared with the placebo-treated control group, and should be considered as a potential drug to be developed for the treatment of AP. There are, however, limitations to the present study, and the underlying mechanisms of AP should be investigated further in future research.

## Figures and Tables

**Table I:** Patient characteristics prior to ITCM treatment.

A, Male patients			

Characteristics	Experiment group	Control group	P-value
Cases (n)	32	32	>0.05^a^
Age (years)	50.5±4.9	50.8±4.8	>0.05^b^
BMI (kg/m^2^)	25.7±6.1	26.0±6.7	>0.05^b^
Daily calorie intake (kcal)	2255±377	2188±409	>0.05^b^
AP classification (n)			
Mild	18	17	>0.05^a^
Moderate	8	8	>0.05^a^
Severe	6	7	>0.05^a^

B, Female patients			

Characteristics	Experiment group	Control group	P-value

Cases (n)	18	18	>0.05^a^
Age (years)	46.9±6.7	48.1±6.9	>0.05^b^
BMI (kg/m^2^)	25.1±5.8	25.7±5.5	>0.05^b^
Daily calorie intake (kcal)	2033±249	2099±276	>0.05^b^
AP classification (n)			
Mild	8	8	>0.05^a^
Moderate	5	5	>0.05^a^
Severe	5	5	>0.05^a^

P-value calculated with the

a t-test and

b χ^2^ test.

ITCM, integrated traditional Chinese medicine; BMI, body mass index; AP, acute pancreatitis.

**Table II:** Comparison of clinical characteristics for AP patients prior to and following ITCM treatment.

	Before treatment			After treatment		
		
Clinical characteristic	Experiment group	Control group	P-value	Experiment group	Control group	P-value
Upper abdominal pain (n)	50	50	>0.05^a^	4	47	<0.05^a^
Findings of ultrasonography (n)	50	50	>0.05^a^	10	48	<0.05^a^
Sudden and sharp cutting pains in upper abdomen (n)	50	50	>0.05^a^	8	47	<0.05^a^
Abdominal distension (n)	50	50	>0.05^a^	6	49	<0.05^a^
Dry and bitter taste (n)	50	50	>0.05^a^	3	46	<0.05^a^
Coated and dry tongue or burned black tongue (n)	50	50	>0.05^a^	5	48	<0.05^a^
Forceful sphygmus (n)	50	50	>0.05^a^	5	47	<0.05^a^
Diffuse enlargement of the pancreas (n)	50	50	>0.05^a^	8	48	<0.05^a^
White blood cells (cells/ml)	1.51×10^5^	1.53×10^5^	>0.05^b^	8.51×10^4^	1.50×10^5^	<0.05^b^
	±42×10^4^	±41×10^4^		±3.58×10^4^	±40×10^4^	
Neutrophils (%)	68.2±5.2	68.8±5.5	>0.05^b^	55.7±4.7	67.3±5.8	<0.05^b^
Lipase (pg/ml)	326±76	318±81	>0.05^b^	207±57	320±71	<0.05^b^
Serum amylase (IU/l)	965±307	952±297	>0.05^b^	709±286	943±255	<0.05^b^
Urine amylase (IU/l)	1876±325	1866±298	>0.05^b^	1054±233	1834±311	<0.05^b^

P-value calculated by the

a t-test and

b χ^2^ test.

AP, acute pancreatitis; ITCM, integrated traditional Chinese medicine.
